# Optimisation of laboratory methods for whole transcriptomic RNA analyses in human left ventricular biopsies and blood samples of clinical relevance

**DOI:** 10.1371/journal.pone.0213685

**Published:** 2019-03-14

**Authors:** Kerrie L. Ford, Maryam Anwar, Rachael Heys, Eltayeb Mohamed Ahmed, Massimo Caputo, Laurence Game, Barnaby C. Reeves, Prakash P. Punjabi, Gianni D. Angelini, Enrico Petretto, Costanza Emanueli

**Affiliations:** 1 National Heart and Lung Institute, ICTEM, The Hammersmith Hospital, Imperial College London, London, United Kingdom; 2 Bristol Heart Institute, School of Translational Health Sciences, University of Bristol, Bristol, United Kingdom; 3 Clinical Trials and Evaluation Unit, Bristol Trials Centre, Bristol Medical School, University of Bristol, Bristol, United Kingdom; 4 University Hospitals Bristol NHS Foundation Trust, Bristol, United Kingdom; 5 MRC London Institute of Medical Sciences, The Hammersmith Hospital, Imperial College London, London, United Kingdom; 6 Imperial College Healthcare NHS Trust, London, United Kingdom; 7 Duke-NUS Medical School, Singapore, Republic of Singapore; Goethe-Universitat Frankfurt am Main, GERMANY

## Abstract

This study aimed to optimise techniques for whole transcriptome and small RNA analyses on clinical tissue samples from patients with cardiovascular disease. Clinical samples often represent a particular challenge to extracting RNA of sufficient quality for robust RNA sequencing analysis, and due to availability, it is rarely possible to optimise techniques on the samples themselves. Therefore, we have used equivalent samples from pigs undergoing cardiopulmonary bypass surgery to test different protocols for optimal RNA extraction, and then validated the protocols in human samples. Here we present an assessment of the quality and quantity of RNA obtained using a variety of commercially-available RNA extraction kits on both left ventricular biopsies and blood plasma. RNA extraction from these samples presents different difficulties; left ventricular biopsies are small and fibrous, while blood plasma has a low RNA content. We have validated our optimised extraction techniques on human clinical samples collected as part of the ARCADIA (Association of non-coding RNAs with Coronary Artery Disease and type 2 Diabetes) cohort study, resulting in successful whole transcriptome and small RNA sequencing of human left ventricular tissue.

## Introduction

Despite improvements in coronary revascularisation techniques, ischaemic heart disease (IHD) remains a leading cause of morbidity and mortality in more economically developed countries,[1] and patients are at risk of developing post-intervention complications.[2] Type 2 diabetes mellitus (T2DM), which is projected to affect 592 million people globally by 2035,[3] is a major risk factor both for IHD and for severe acute post-surgical complications following coronary intervention, such as acute kidney injury. New therapeutic targets and better non-invasive biomarkers are needed to improve IHD treatment and the post-revascularization outcomes, particularly in the context of comorbidities such as type 2 diabetes.

Translational research provides the unique possibility to understand transcriptomic changes in the diseased human heart and how this is reflected in factors released into the circulation, facilitating identification of new targets for therapeutic intervention and as non-invasive biomarkers of cardiac conditions. The increasing sophistication of sequencing technology and big data analysis is starting to reveal the dynamic transcriptomic changes underlying disease processes. Non-coding RNAs (ncRNAs), particularly microRNAs (miRNAs), have received much attention for their potential as therapeutic targets, due to their role as regulators of gene expression, and notably the ability of individual ncRNA to act as a network hub, regulating multiple transcripts. In particular, the discovery that miRNAs and other ncRNAs can be detected in the circulation, protected from degradation by RNases due to their encapsulation in extracellular vesicles (EVs),[4–6] or as part of protein[7] or lipoprotein complexes,[8] has increased the interest in developing miRNAs as extracellular clinical biomarkers.[9–12] According to pioneering studies in small sample populations, miRNAs have the potential to enable early detection of the impact of risk factors, acute interventions and disease conditions on the heart, and may be predictive of outcomes.[13, 14]

Understanding the systemic nature of cardiometabolic diseases requires investigation of multiple affected tissues, while the discovery and development of novel biomarkers is best done in peripheral accessible biofluids. Given the diverse sample types, one challenge of this type of study is to optimise analysis methods for each sample type while ensuring the methods themselves aren’t introducing bias. A cautionary example of the effects of methodology on discovery is given by Kim *et al*, who retracted their 2011 Molecular Cell paper after discovering that their TRIzol-based RNA extraction method selectively lost miRNAs with low GC-content when isolating RNA from low cell numbers, leading them to draw incorrect conclusions.[15]

In recent years several groups have published excellent systematic analyses of different RNA extraction protocols on plasma and serum, although as yet there is no consensus as to the optimal protocol.[16–28] It is challenging to isolate RNA from these samples due to the low starting concentration of RNA in biofluids, and the abundance of both RNases and PCR inhibitors in the samples. Equally, samples from different tissues present their own specific challenges; in the case of left ventricular biopsies, the small size and fibrous nature of the tissue present significant barriers to obtaining high-quality RNA. The dynamic and sensitive nature of RNA makes it imperative that discovery, validation and translation studies are carried out with rigorous and systematic methodology,[29–31] especially in the context of clinical studies where underlying patient variability generates a high signal-to-noise ratio.

Here we present methodological work with findings that may be useful for others working on similar clinical samples. Before beginning work with human samples we have tested 6 commercially-available RNA extraction kits on left ventricular biopsies and blood plasma from 3 pigs undergoing cardiopulmonary bypass, assessing yield, quality, and RT-qPCR performance. The results from this were validated on equivalent human surgical samples, which were successfully used for RNAseq and miRNA RT-qPCR screening. We highlight the importance of comprehensive validation of basic methods used when preparing samples for high-throughput experiments to ensure data are representative and biologically valid.

## Materials and methods

### Study design

ARCADIA is an observational prospective cohort study developed between two-centres, the Bristol Royal Infirmary (University Hospitals Bristol NHS Foundation Trust) and the Hammersmith Hospital (Imperial College Healthcare NHS Trust). Written informed consent was obtained from all patients. All human samples were obtained in accordance with the principles of the Declaration of Helsinki. The study was reviewed and approved by the National Research Ethics Service Committee London–Fulham (date of approval, 20 Dec 2013; reference 13/LO/1687).

Further details of the ARCADIA study are provided in the supplemental methods.

### Pigs

Samples were obtained from 18–24 kg female White Landrace pigs (*Sus scrofa domesticus*) undergoing non-recovery cardiopulmonary bypass surgery. These pigs were enrolled in a separate study, and the samples obtained were surplus tissue and biofluids taken immediately prior to termination. All animal experiments were performed in accordance with the UK Animals (Scientific Procedures) Act 1986, amended 2012, conducted under the UK Home Office licence 70/8975 and were approved by the University of Bristol Animal Welfare and Ethical Review Body.

### Sample processing

Samples from pigs and from human patients were collected and stored using identical methods.

After establishment of cardio-pulmonary bypass, left ventricular (LV) biopsies were taken from the apex of the heart using a biopsy needle; biopsies were 14 gauge in core diameter and 3–5 mm in length. After collection, the LV biopsies were placed immediately into RNA*later* (Ambion) and incubated overnight at 4°C. The RNA*later* was then removed and the sample frozen at -80°C.

Peripheral blood was obtained from the arterial line, collected into a 5 mL sodium citrate vacutainer. Whole blood was centrifuged at 2240 *g* for 10 minutes at room temperature. The plasma supernatant was immediately removed to a fresh tube without disturbing the buffy coat, then centrifuged again at 2240 *g* for 10 minutes at room temperature. The supernatant was removed to a fresh tube and stored at -80°C.

### RNA extraction

RNA isolation from pig LV biopsies was performed using four commercially available kits: A) RNAqueous-micro (Ambion, now Invitrogen, AM1931); B) *mir*Vana (Ambion, now Invitrogen, AM1560); C) miRCURY tissue (Exiqon, now Qiagen, 300115); D) miRNeasy micro (Qiagen 217084). Biopsies were weighed, then homogenised at 4°C in the recommended volume of lysis buffer using a Bertin minilys with ceramic beads, 4 x 30 second bursts at maximum speed with 5 minutes incubation on ice between each burst. Following homogenization, lysates were centrifuged at 300 *g*, 5 minutes, 4°C to minimize foam, then removed to a fresh tube. Thereafter, the manufacturer’s protocol was followed exactly in one set of extractions, while in a parallel set of extractions the protocol was modified to include a proteinase K digestion step; samples were incubated with 200 μg/mL proteinase K (Invitrogen AM2546) for 15 minutes at 55°C. For the miRCURY tissue kit the manufacturer’s proteinase K digestion protocol was followed. All products were eluted in the recommended volume of RNase-free water and stored at -80°C in low-bind tubes.

RNA extraction from human LV biopsies was performed using the *mir*Vana kit (AM1560) following the manufacturer’s protocol, with the exception that samples were eluted in 50 μL nuclease-free water.

RNA isolation from 200 μL pig plasma (frozen at -80°C and thawed once) was performed using four commercially available kits: A) miRNeasy serum/plasma (Qiagen 217184); B) *mir*Vana (Ambion, now Invitrogen, AM1560); C) miRCURY biofluids (Exiqon, now Qiagen, 300112); D) Plasma/serum RNA purification mini kit (Norgen Biotek 217084). In one set of extractions the manufacturer’s protocols were followed, while in a parallel set the protocol was modified to include glycogen (Thermo Fisher RNA grade glycogen R0551) as a co-precipitant at a final concentration of 0.4 μg/μL. All plasma RNA extractions included 10 μL of 5 fmol/μL synthetic cel-miR-39-3p (Qiagen 219610) spiked in to the relevant lysis buffer as an exogenous control for isolation efficiency.[11]

RNA isolation from 200 μL human plasma (frozen at -80°C and thawed once) was performed using the Qiagen miRNeasy serum/plasma kit (217184), following the manufacturer’s protocol, with the addition of glycogen as a co-precipitant to a final concentration of 0.4 μg/μL. Instead of using the cel-miR-39-3p spike-in, we used the Exiqon UniSp2/UniSp4/UniSp5 spike-ins required for subsequent analysis of the RNA using the miRCURY LNA Universal RT microRNA PCR System human panels I and II (*vide infra*).

### RNA quality and quantity measurements

LV RNA was quantified and assessed for purity by spectrophotometry, using a NanoDrop (Thermo Scientific). RNA integrity was assessed using a Bioanalyzer 2100 (Agilent) Nano chip and Small RNA chip.

### RT-qPCR

Stem-loop reverse transcription primers and PCR primers/hydrolysis probes were used for data on pig plasma (Applied Biosystems TaqMan MicroRNA assays; cel-miR-39-3p 000200, hsa-miR-15b-5p 000390, hsa-miR-16-5p 000391, hsa-miR-21-5p 000397, hsa-miR-24-3p 000402, hsa-miR-92a-3p 000431, hsa-miR-103a-3p 000439, hsa-miR-126-3p 002228). Reverse transcription on pig plasma RNA was performed using the TaqMan MicroRNA Reverse Transcription Kit (Applied Biosystems 4366596) with a modified protocol to include heparinase I (from *Flavobacterium heparinum*, Sigma-Aldrich H2519) digestion, since residual heparin administered during surgery may interfere with downstream reactions.[32] Briefly, template RNA was incubated with RNase inhibitor, reaction buffer and heparinase I (to a final concentration of 0.03 U/μL) for 60 minutes at 25°C. dNTPs, primer, nuclease-free water and reverse transcriptase were then added, and the remainder of the reverse transcription reaction carried out according to the manufacturer’s instructions.

qPCR reactions on pig plasma were carried out in triplicate using the TaqMan Universal PCR Master Mix, no AmpErase UNG (Applied Biosystems 4324018) master mix, using a QuantStudio 6 Flex Real-Time PCR System (Applied Biosystems) with the manufacturer’s thermocycling protocol. The manufacturer’s instructions were scaled to a final PCR volume of 12 μL. Raw C_q_ values were used for method comparisons.

RT-qPCR on human plasma was performed using the miRCURY LNA Universal RT microRNA PCR System human panels I and II v4.0 (Exiqon 203600), with the Universal cDNA Synthesis Kit II (Exiqon 203301) including the UniSp6 and cel-miR-39-3p (Exiqon 203203) spike-ins, and ExiLENT SYBR Green Master Mix (Exiqon 203403). C_q_ values were calibrated using the UniSp3 inter-plate calibrator, with an upper threshold of C_q_ = 37 applied.

### RNA sequencing

RNA extracted from human left ventricular tissue was digested with DNase I (Invitrogen DNA-*free* DNA removal kit AM1906). 20 ng digested RNA was depleted of rRNA (NEBNext rRNA depletion kit E6350L) and libraries were constructed using the NEBNext Ultra II Directional RNA Library Prep Kit for Illumina (E7760S). The libraries were sequenced on an Illumina HiSeq 2500 using paired-end 100 bp reads and at a depth of ~60 million reads per sample.

The quality of sequenced data was assessed using the program FASTQC.[33] Samples from both the libraries that passed the QC step were aligned to the human genome (hg19) using the aligner STAR[34] and raw read counts were generated using the R package Rsubread.[35, 36] Annotations were carried out using Ensembl GRCh37 (v75) and miRBase v20. Raw read counts were normalised using Trimmed Mean of M-values (TMM) method.[37] miRNAs that were expressed at very low levels (raw_count<5 in > = 25% of the samples within each patient category) were screened out and the remaining miRNAs were ranked from high to low-expressed by calculating an average of their normalised counts across all patient categories.

## Results

### RNA isolation from pig left ventricular biopsies

We tested four isolation kits for extraction of total RNA, including small RNA, from pig LV biopsies. Like human samples, these samples are challenging to isolate sufficient quantity and quality of RNA for RNAseq due to their small size and fibrous nature. All kits tested were described by the manufacturer as being optimised for isolation of small RNAs, while the miRNeasy micro and RNAqueous-micro are intended for isolation of RNA from small samples, and the miRCURY tissue specifies its suitability for isolation from fibrous tissues. The miRNeasy micro and *mir*Vana protocols involve a phenol-chloroform isolation followed by a column-based clean up, while the RNAqueous-micro and miRCURY tissue are phenol-free and rely on spin column chromatography to recover RNA. Three of the kits were tested with and without an additional proteinase K digestion step, as proteinase K is reported to improve recovery of RNA.[38] The fourth kit, miRCURY tissue, includes a proteinase K digestion as part of the protocol, therefore this kit was not tested in the absence of proteinase K.

**Fig 1:**
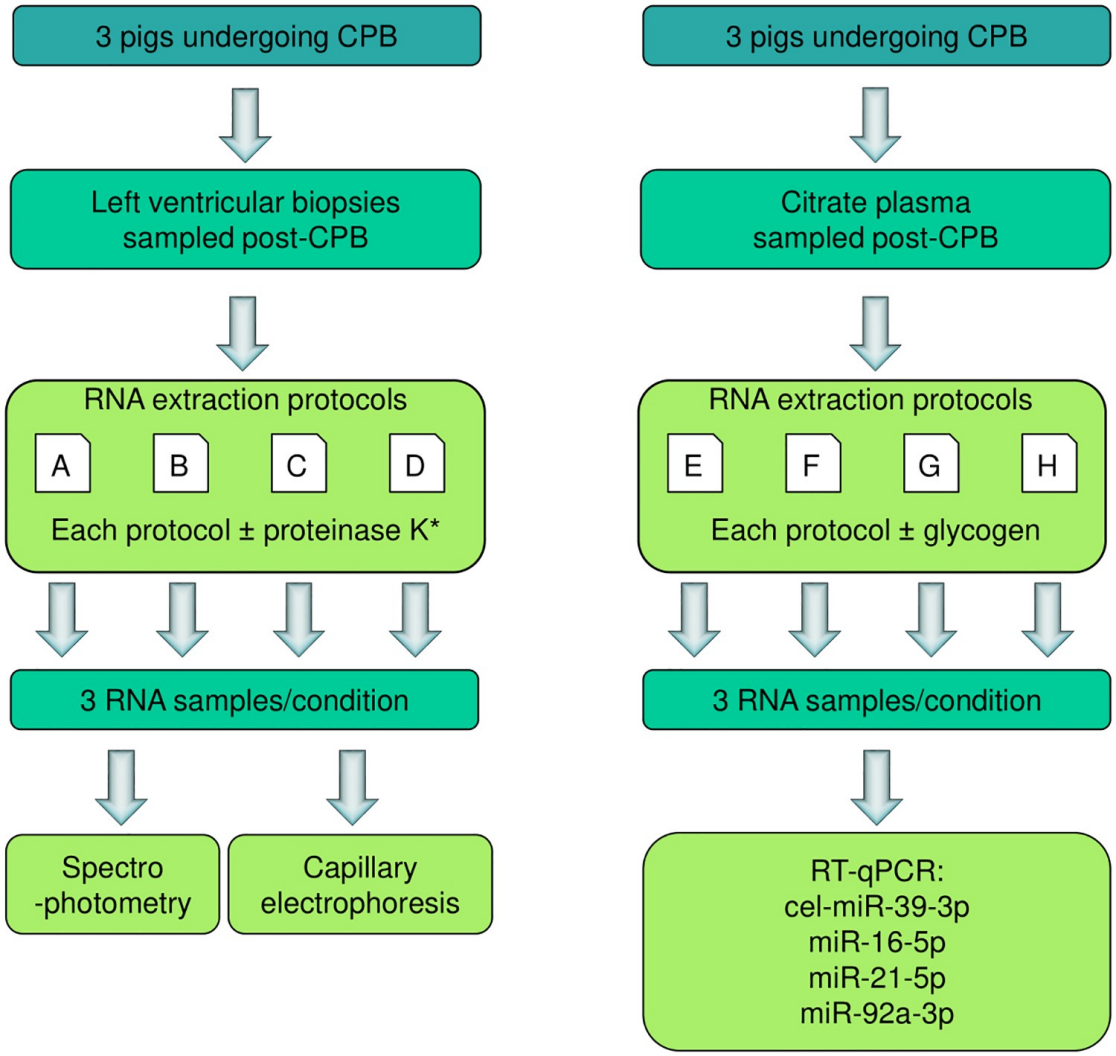
Summary of experimental design for Figs 2, 3 and 4. (A) Left ventricular biopsies were taken from three pigs undergoing cardio-pulmonary bypass (CPB). 7 biopsies were taken per pig. RNA was isolated from the biopsies using four commercially available kits; RNAqueous micro, *mir*Vana, miRCURY tissue, miRNeasy micro. Three kits (RNAqueous micro, *mir*Vana, miRNeasy micro) were tested with and without an additional proteinase K digestion step, giving 7 protocols in total. The miRCURY tissue protocol includes a proteinase K digestion, therefore this kit was not tested without this. RNA samples were characterised by spectrophotometry and capillary electrophoresis. (B) Blood samples were taken from three pigs undergoing CPB and processed to plasma. RNA was isolated from the plasma using four commercially-available kits; miRNeasy serum/plasma, *mir*Vana, miRCURY biofluids, Norgen Biotek plasma/serum. Each kit was tested with and without glycogen as a co-precipitant, giving 8 protocols in total. RNA samples were evaluated by RT-qPCR for the exogenous spike-in cel-miR-39-3p, and endogenous miRNAs miR-16-5p, miR-21-5p, miR-92a-3p.

**Fig 2:**
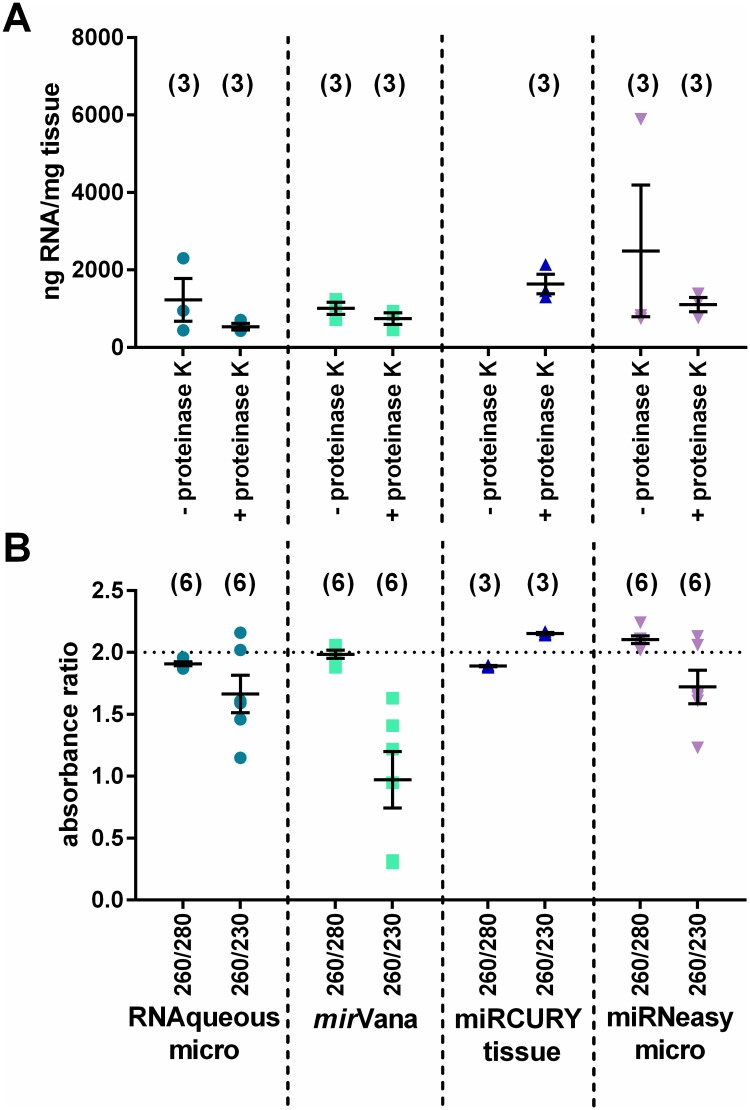
Spectrophotometry analysis of pig LV biopsy RNA. RNA extracted according to each manufacturer’s protocol, with the addition of a proteinase K digestion in half the samples. n = 3 biopsies/experimental condition, mean±SEM. *N*.*B*. Exiqon miRCURY tissue protocol requires proteinase K, therefore no–proteinase K condition was done with this kit. (A) RNA yield, assessed as ng RNA recovered per mg input tissue, was not significantly different between the protocols (Kruskal-Wallis test with Dunn correction), and additional proteinase K digestion in the RNAqueous micro, *mir*Vana and miRNeasy micro protocols did not significantly improve RNA yield (Kruskal-Wallis test with Dunn correction). (B) 260/280 and 260/230 ratios provide an assessment of the purity of the RNA. Ratio <2.0 indicates possible contamination with protein, phenol or salts. All protocols produced an acceptable 260/280 ratio. RNA extracted using the *mir*Vana protocol had low 260/230 ratios, indicating probable phenol or guanidine carryover. Numbers in brackets indicate the number of samples in each group.

Initial tests were performed on LV biopsies from pigs undergoing cardiopulmonary bypass surgery. Each combination of kit ± proteinase K was tested on one biopsy from each of three pigs (Fig 1). The eluted RNA was initially quantified based on absorbance in the UV-visible spectrum using a NanoDrop (Fig 2). The four kits tested have different elution volumes, and biopsies varied in mass from 0.2–17.5 mg, reflecting the variation in human clinical samples, therefore to directly compare kit performance total eluted RNA is expressed relative to initial biopsy mass. The proteinase K digestion step did not significantly increase the RNA yield in any of the three kits tested ± proteinase K (Fig 2A). In each case, the mean yield following proteinase K digestion was lower, although the differences were not statistically significant (Kruskal-Wallis test with Dunn correction for multiple comparisons).

Spectrophotometry also gives information about the purity of an RNA sample, based on the ratios of absorbance at 260 nm (nucleic acid peak absorbance) to 280 nm and 230 nm (peak absorbances of other contaminants including protein, guanidine and phenol). A 260/280 of ~2.0, and 260/230 of ~2.0–2.2 indicate good RNA purity. All the protocols tested had acceptable 260/280 values (Fig 2B), while the *mir*Vana kit had a 260/230 <2.0, indicating probable phenol and/or guanidine carryover. The presence of these contaminants may inhibit downstream reactions, therefore the sensitivity of the intended use of the RNA should be taken into consideration when choosing the best method for RNA extraction.

**Fig 3:**
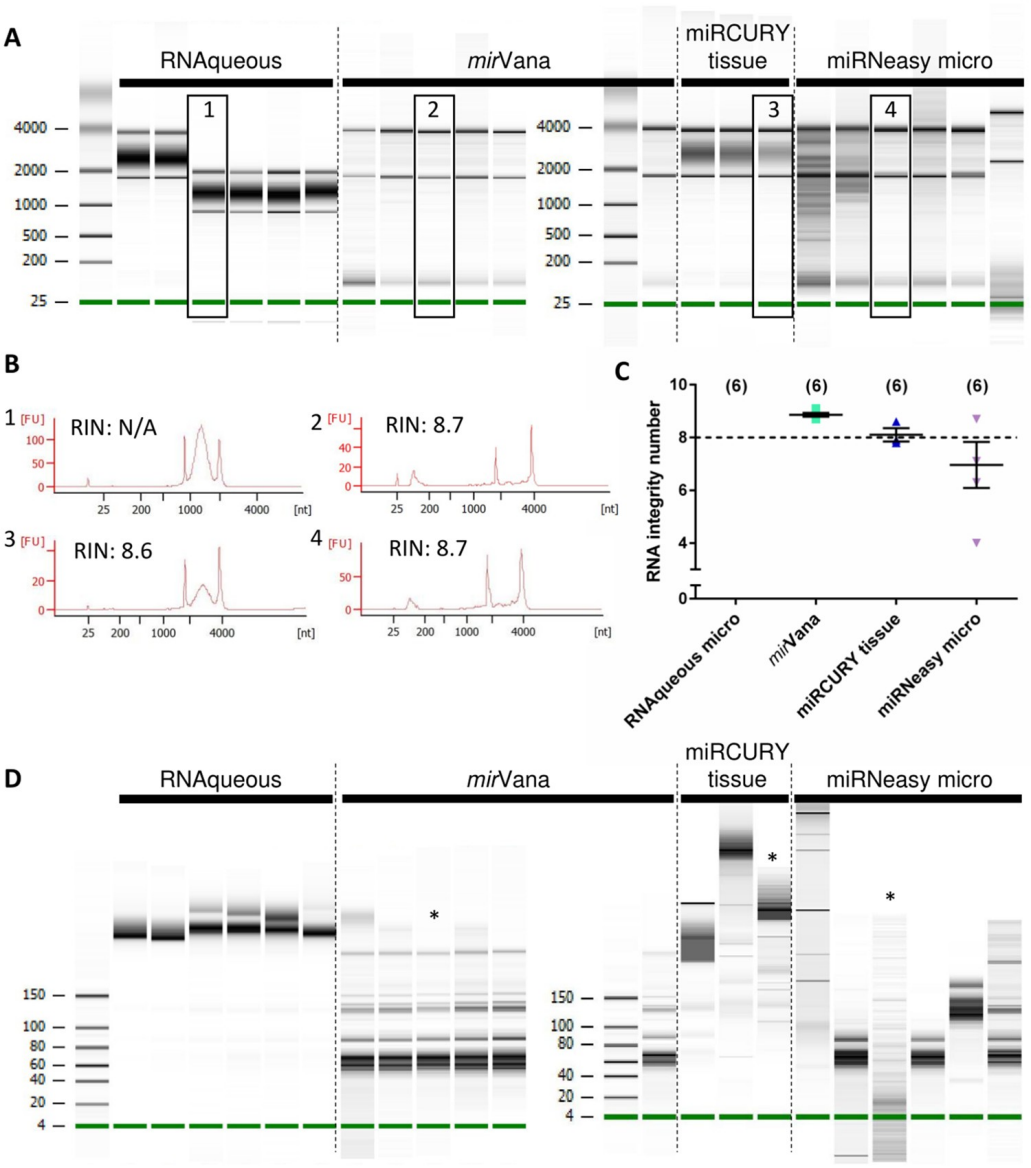
Capillary electrophoresis analysis of pig LV biopsy RNA. **(**A) Bioanalyzer nano chip traces of all tested samples. Inspection of electropherograms provides additional information to assess RNA quality and protocol performance. (B) Representative traces from each protocol with respective RIN values. Numbers 1–4 correspond to numbered boxes in A. Note that trace 3 has a high RIN despite a large smear in the gel. (C) Summary of RIN values of all the samples tested (mean±SEM). The RNAqueous protocol had undetectable RINs. The *mir*Vana and miRCURY tissue protocols gave consistently high RIN values, while despite high average RIN, the samples from the miRNeasy micro protocol are more variable. Numbers in brackets indicate the number of samples in each group. (D) Bioanalyzer small RNA chip traces for each kit. Although the RIN of samples marked * were similar (8.6–8.7), the spectrum of small RNAs recovered is highly heterogeneous.

Due to the ubiquitous presence of RNases in the environment, RNA is vulnerable to degradation, which will adversely affect downstream experiments, therefore RNA integrity must be assessed by electrophoresis. The Bioanalyzer system provides on-chip RNA electrophoresis; analysis using Nano or Pico chips includes calculation of an RNA Integrity Number (RIN) and RNA concentration, while the Small RNA chip permits examination of RNA in the range 6–150 nt. LV biopsy RNA samples were analysed using both Nano and Small RNA chips. Consistently high (>8) RIN values were obtained using the *mir*Vana and miRCURY tissue kits (Fig 3A), however inspection of the electropherogram traces revealed a smear in the 2500–3500 nt range for the samples isolated with the miRCURY tissue kit (Fig 3A) that is not reflected in the RIN values. The miRNeasy micro protocol gave variable RIN values (Fig 3C), while the RNAqueous-micro kit had undetectable RINs in all samples, and inspection of the electropherograms reveals an unexplained shift in mobility for 4/6 samples (Fig 3A). RNA concentrations measured with the Bioanalyzer Nano chip showed similar results to the NanoDrop measurements (Fig 2A cf. S1 Fig).

The small RNA chip shows that the RNAqueous-micro and miRCURY tissue kits consistently failed to recover RNAs in the miRNA range from these samples. The miRNeasy micro and *mir*Vana kits more reliably recovered small RNAs, however it is worth noting that a high RIN score from the Nano chip does not necessarily correspond to good small RNA recovery, therefore if small RNAs are of interest it is essential to run both assays during validation (Fig 3D).

### RNA isolation from pig plasma

We also tested four isolation kits for extraction of RNA from plasma. Three of the kits were described by the manufacturer as being optimised for recovery of RNA from ‘biofluids’, or specifically serum/plasma. Initial tests were performed on citrate plasma from pigs undergoing cardiopulmonary bypass surgery. 200 μL plasma from each of three pigs were used to test each kit, with and without the use of glycogen as a co-precipitant (Fig 1). Glycogen helps to precipitate nucleic acids and is particularly valuable in recovering low-yield RNA.[17, 21, 39] In each case, synthetic cel-miR-39 was spiked-in to the initial lysis solution as an exogenous control.[11]

**Fig 4:**
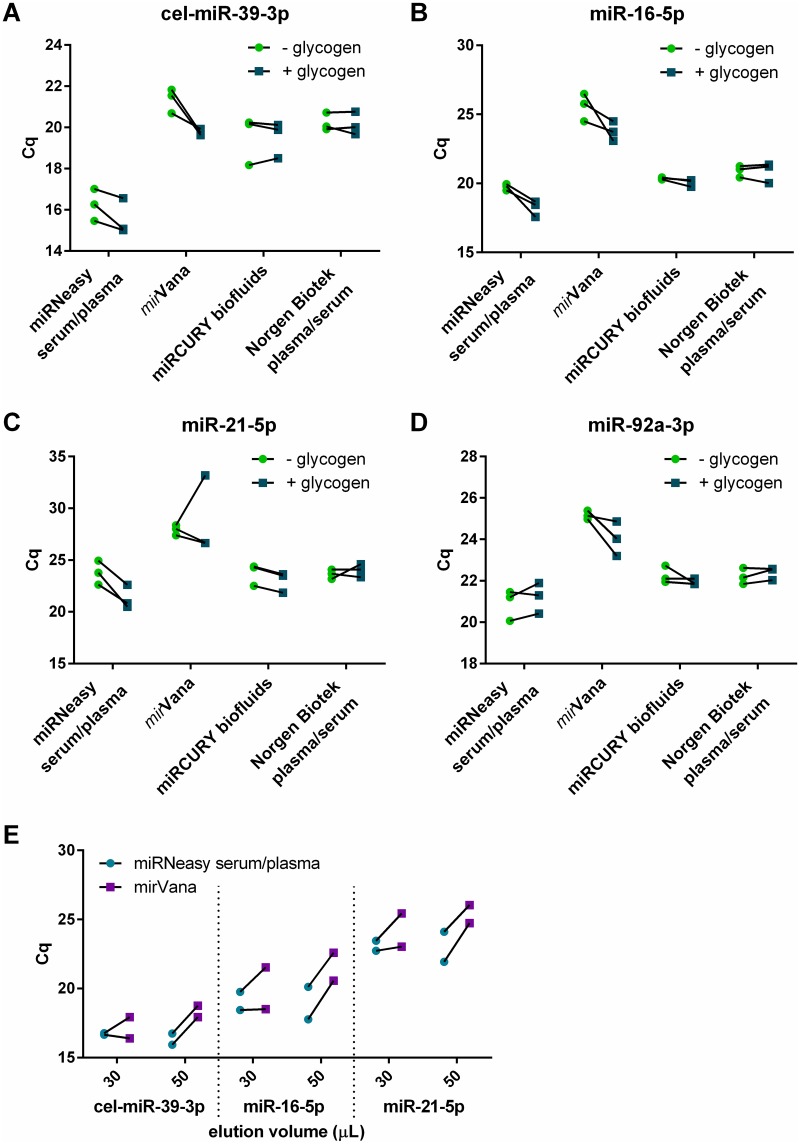
Evaluating plasma RNA yield using RT-qPCR for miRNAs. As biofluid RNA is below the detection limit of standard quantification tools, all RT-qPCRs were performed using 1.5 μL RNA extracted from 200 μL plasma. Lines indicate paired samples i.e. aliquots of plasma from the same blood draw. (A) RT-qPCR for the spike-in cel-miR-39-3p using the four kits with and without glycogen. (B-D) RT-qPCR for three endogenous miRNAs isolated using the four kits with and without glycogen. (E) The miRNeasy serum/plasma protocol recommends elution in 14 μL, compared with 100 μL for the *mir*Vana protocol. To control for effects of this, RNA extractions were performed with both protocols, eluting in 30 μL or 50 μL. RT-qPCR for cel-miR-39-3p, hsa-miR-16-5p and hsa-miR-21-5p were performed to evaluate the different elution volumes.

Biofluids typically contain very low concentrations of RNA, below the current detection limit of spectrophotometry or electrophoresis.[30, 40] Therefore, to compare the protocols apparent RNA recovery was assessed by RT-qPCR for the exogenous control cel-miR-39 (Fig 4). Addition of glycogen lowered the C_q_ in all samples extracted using the miRNeasy serum/plasma and *mir*Vana kits (mean 0.69 and 1.59 C_q_ respectively), although this was only significant for the *mir*Vana kit (p = 0.011, t test with Holm-Sidak correction for multiple comparisons). Glycogen did not appear to affect C_q_ values for RNA extracted with the other two methods.

To test recovery of endogenous miRNAs, we screened a panel of 7 functionally important miRNAs that are predicted to be conserved between humans and pigs, known to be present in human heart and circulation and with relevance to cardiovascular function and disease. Briefly, miR-15b-5p and miR-16-5p are upregulated in cardiomyocytes during cardiac ischaemia and are involved in angiogenesis,[41] miR-21-5p is highly expressed throughout the cardiovascular system, promotes fibrosis and is dysregulated in the heart and vasculature in disease states,[42–44] miR-24-3p is expressed in cardiomyocytes and endothelial cells and has been highlighted as a potential circulating biomarker,[45–47] miR-92a-3p is expressed in endothelial cells and is involved in atherosclerosis,[44] miR-103a-3p circulates in plasma and may associate with acute myocardial infarction,[48] and miR-126-3p is expressed in endothelial cells and is involved in angiogenesis and atherosclerosis, [44, 49]

Preliminary screening showed that the tested miRNAs demonstrated robust amplification (S3 Fig). Finally, all samples were screened for miR-16-5p, miR-21-5p and miR-92a-3p, which represented a range of expression levels and GC content (miR-16-5p = 45.5%, miR-21-5p = 36.4%, miR-92a-3p = 52.2% GC). As with cel-miR-39, miR-16-5p and miR-21-5p recovery was enhanced by co-precipitation with glycogen, with the effect seen most strongly for the miRNeasy serum/plasma and *mir*Vana kits (Fig 4B and 4C), however the results for miR-92a-3p were equivocal (Fig 4D). It has been reported that miRNAs with low GC content are recovered poorly from low RNA concentration samples.[15] It is possible that the improved recovery due to co-precipitation with glycogen has a bigger effect on recovery of low GC content miRNAs such as miR-21-5p than high GC content miRNAs such as miR-92a-3p, although a more thorough analysis would be required to demonstrate this conclusively.

In all cases, the miRNeasy serum/plasma kit with added glycogen had the lowest C_q_ values. This may be attributable to the low elution volume (14 μL) producing a more concentrated sample, rather than superior total recovery. Therefore, we compared the miRNeasy serum/plasma kit with the *mir*Vana kit, using elution volumes of 30 and 50 μL. The *mir*Vana kit was chosen for this comparison because i) this kit performed best with the LV biopsy RNA (*vide supra*) and ii) this kit has the most comparable protocol to the miRNeasy serum/plasma. Fig 4E shows that even with equal elution volumes, the miRNeasy serum/plasma kit resulted in significantly lower C_q_ values for the tested miRNAs (p = 0.0015, Wilcoxon matched pairs signed rank test).

### RNA isolation from human left ventricular biopsies

The protocol validation on the pig biopsies suggested that overall the *mir*Vana kit produced the best RNA isolation for this sample type, with consistent yield, high RIN and reasonable recovery of small RNAs, although the low 260/230 ratios remain a factor to consider. One additional consideration is that this kit recommends elution in 100 μL water; with small tissue samples this can result in a sample too dilute for some downstream applications. Although precipitation protocols can allow concentration of RNA samples, we were reluctant to introduce further processing steps that may introduce additional variability. Instead, reducing the elution volume to 30 or 50 μL produced a more concentrated preparation without appreciable loss of RNA (100 μL vs 30 μL p = 0.2359, Kruskal-Wallis test with Dunn’s correction for multiple comparison, S2 Fig).

We used this protocol to isolate RNA from 96 human LV biopsies (0.1–6 mg) collected as part of the ARCADIA study. 92/96 biopsies had a RIN>7, and 71/96 had RIN>8, which is considered high enough quality for demanding applications such as RNAseq (Table B in S1 File). Two RNA sequencing libraries were created, one for small RNAs and one for whole transcriptome analysis. Both libraries passed QC and were successfully sequenced, validating our RNA extraction method as suitable for RNA sequencing applications.

The quality of sequenced data was assessed using the program FASTQC.[33] The quality of the small RNA library was found to be very good with mean quality Phred scores of over 30. There were no over-represented sequences. However, adaptor content was found which was trimmed before any subsequent analysis. The library size was estimated at 20 million reads and read length between 50 and 60 bp after removal of adaptors. The quality of sequenced data for the whole transcriptome library was equally good (mean quality Phred scores >30), with the exception of one sample, which was found to have unusually high rRNA contamination. This sample was therefore excluded from downstream analyses. All the other samples showed no over-represented sequences or adaptor content. The library size was estimated at 70 million paired-end reads with a read length of 100 bp.

From the small RNA sequencing library, we were able to retrieve the 7 miRNAs that were screened in the pig sample validation experiments (miR-16-5p, miR-126-3p, miR-24-3p, miR-103a-3p, miR-21-5p, miR-92a-3p, miR-15b-5p). All the miRNAs ranked very high among the most expressed miRNAs (S4 Fig).

### RNA isolation from human plasma

Recovery of miRNAs from pig plasma was consistently highest using the Qiagen miRNeasy micro kit with additional glycogen co-precipitation, therefore this protocol was used for further biofluids RNA extractions from human clinical samples. RNA extracted from pre-operative peripheral plasma, intra-operative coronary sinus and ascending aorta plasma from patients enrolled in the ARCADIA study was screened for 752 miRNAs using human panels I and II (Exiqon). To verify the success of this method, the expression of the miRNAs screened for in the pig plasma was evaluated in the human pre-operative peripheral plasma samples (S5 Fig). All miRNAs were robustly expressed.

## Discussion and conclusions

Our results indicate that for technically demanding and costly applications such as RNA sequencing or other high-throughput screening methods, it is critical to validate the RNA extraction method for each sample type, as no single method is optimal for all. To the best of our knowledge, few similar systematic analyses have been performed on other cardiovascular clinical tissue sample types. Ip *et al* compared an organic extraction method with a solid-phase extraction method on archived right atrial appendage samples,[50] however these samples are different in size and composition from the more clinically-relevant left ventricular biopsies obtained in the ARCADIA study.

Given the range in size of biopsies obtained clinically, it must be considered that some protocols may perform differently at the upper or lower limits of tissue mass, with respect to either RNA yield or RNA quality. The between-protocol variability was too high to evaluate this for the pig LV biopsies, however the large number of human samples available provided the opportunity to investigate this for the *mir*Vana protocol. RNA yield per mg tissue was consistent in the range 1–6 mg (linear regression slope -0.0161; S6A Fig). Below 1 mg, an inverse relationship between biopsy mass and yield was observed (S6B Fig); with linear regression the slope was -3.4094, however the relationship was better described by a power function (*y* = 1.503*x*^−0.4715^ least squares fit). While this may indicate that the protocol performs more efficiently on smaller masses of tissue, it is more likely that this reflects the proportionately higher contribution of contaminants to the measured yield. No saturation effect on yield was observed at the upper bound. This is not surprising for this protocol, given the manufacturer’s stated upper limit of 250 mg, however it is one factor that should be considered when evaluating a protocol. No strong relationship between biopsy mass and RIN was observed, although it is notable that those few biopsies that yielded poor-quality RNA were <1 mg (S6C Fig).

Previous studies have compared different protocols for isolating RNA from plasma and serum,[16–28] including the protocols tested here. The findings from these studies were often not in agreement; several found the highest yield/lowest C_q_ values following RT-qPCR with Qiagen kits,[16, 21, 23, 26, 27] while others had better results with Exiqon,[17] Ambion[18] or Machery-Nagel[19, 22, 25] kits. This suggests that other variables are significantly affecting the results. These could be pre-analytical variation in sample handling, or user variation in execution of the kit protocols. Furthermore, other notable findings from these studies include 1. overall yield may not translate to optimal RT-qPCR results,[24] 2. one protocol may have good yield but high variability,[23] 3. the use of carriers/co-precipitants increases yield in some, but not all, protocols,[17, 21, 28] 4. some protocols may recover miRNAs and mRNAs with different efficiencies,[20] 5. miRNA recovery is affected by miRNA GC content, folding energy and relative abundance, and this is protocol-specific.[15, 28]

For large-scale clinical studies, the cost of reagents used to process samples is inevitably an additional consideration. Based on the list price in British pound sterling (£) at the time of writing, for the LV biopsies the kit cost per sample ranges from £6.08 (RNAqueous micro), to £6.94 (miRNeasy micro), to £7.88 (*mir*Vana with phenol). The best-performing kit in our hands is also the costliest, therefore users may need to factor this in to their choice of reagents, although we note that this kit is also available at a reduced cost if the user supplies the phenol. The biofluids-specific kits are more expensive, ranging from £7.62 (miRNeasy serum/plasma) to £8.08 (Norgen Biotek plasma/serum) per sample. While this manuscript was being prepared, Exiqon became part of the Qiagen portfolio, therefore a direct cost-comparison of the miRCURY kits was not possible.

**Fig 5:**
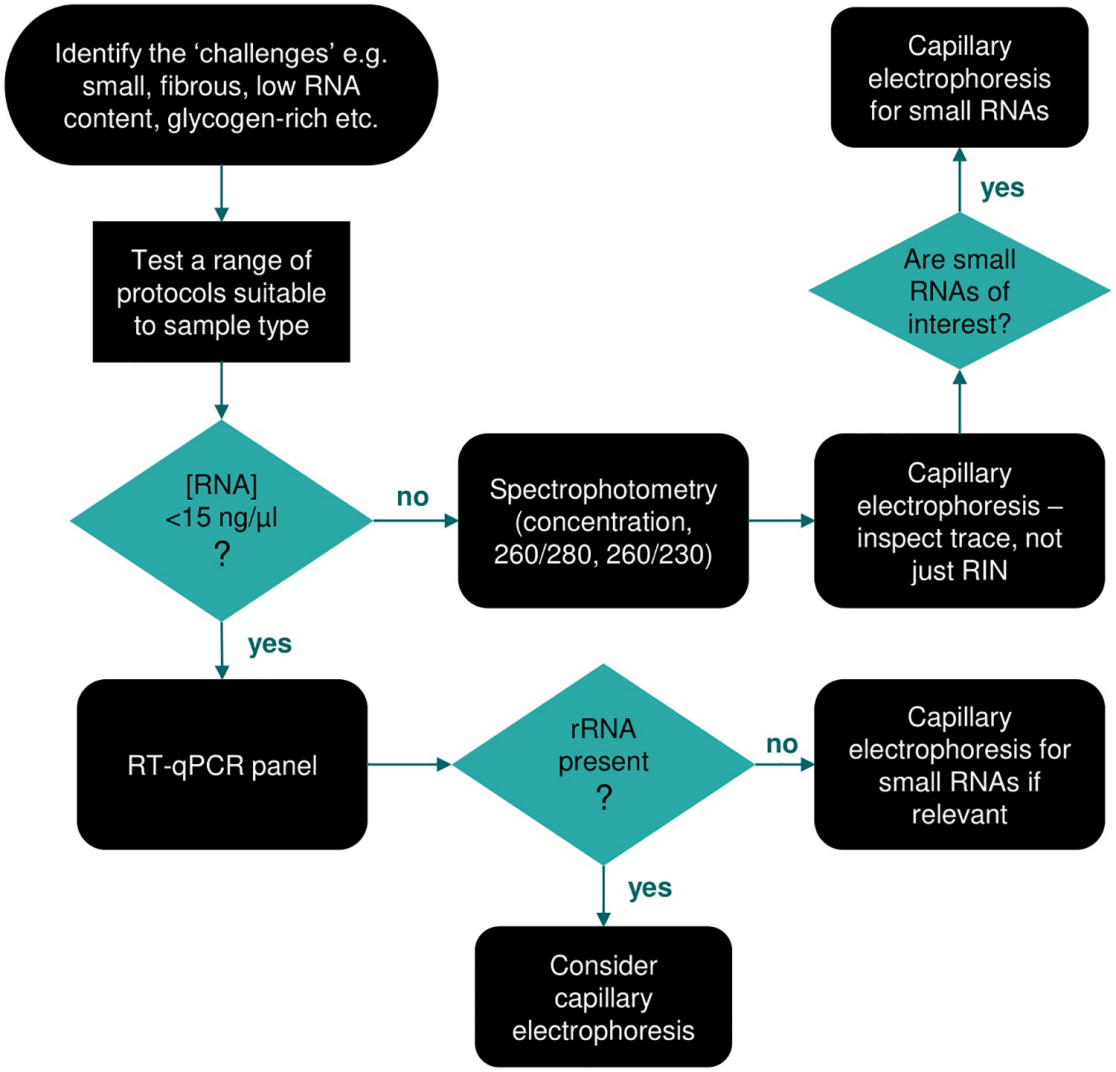
Decision tree for optimising appropriate protocols for RNA extraction from challenging samples.

Based on our experience, we would make the following general recommendations for others working on similar clinical samples (Fig 5). RNA quality should be assessed by both spectrophotometry and capillary electrophoresis where possible, however we would caution that Bioanalyzer results should be treated critically–a high RIN alone may not indicate a successful extraction, as inspection of the electropherograms may indicate problems with the RNA not detected by the RIN calculation. Considering this, we would encourage authors to publish representative Bioanalyzer traces as well as RIN values. Furthermore, if small RNAs are of interest in subsequent analyses, the Small RNA chip should be run, since a high RIN does not necessarily indicate good recovery of small RNAs. For low RNA content samples i.e. most biofluids, where traditional quality control methods are not viable, the recovery of a panel of RNAs of interest should be verified by RT-qPCR. Ideally the panel would be populated with a range of RNAs, both exogenous (spiked-in) and endogenous, where the endogenous species should represent different GC contents and abundances.

## Supporting information

S1 File Supplemental methods and results table. An expanded methods section and Table B in S1 File can be found in the online supplement, “supplemental methods and results table.docx”.
(DOCX)

S1 Fig Capillary electrophoresis quantification of pig LV biopsy RNA. Bioanalyzer Nano chip quantification of LV biopsy RNA was similar to the spectrophotometry quantification presented in Fig 2A. RNA extracted according to each manufacturer’s protocol, with the addition of a proteinase K digestion in half the samples. n = 3 biopsies/experimental condition, mean±SEM. *N*.*B*. Exiqon miRCURY tissue protocol requires proteinase K, therefore no–proteinase K condition was done with this kit. Numbers in brackets indicate the number of samples in each group.
(TIF)

S2 Fig Comparison of elution volumes for the *mir*Vana protocol. The *mir*Vana protocol recommends elution in 100 μL water; with small tissue samples this can result in a sample too dilute for some downstream applications. Reducing the elution volume to 50 or 30 μL produced a more concentrated preparation without appreciable loss of RNA yield, measured by spectrophotometry (p = 0.2359, Kruskal-Wallis test).
(TIF)

S3 Fig miRNA screening in pig plasma RNA. Two randomly selected pig plasma RNA samples were screened for the presence of 7 candidate circulating miRNAs predicted to be conserved between humans and pigs.
(TIF)

S4 Fig The top 35 miRNAs detected in human LV biopsy RNA. The expression levels of the top 35 miRNAs in LV biopsies are plotted as log2 normalised counts. For each plotted miRNA, the box indicates the upper (75%) and lower (25%) quartiles of the counts with a straight black line within the box indicating the median. The whiskers of the box indicate counts that lie outside of this. Circles represent outliers. All candidate circulating miRNAs from pig plasma are also expressed in human LV; only hsa-miR-15b-5p lies outside the top 35.
(TIF)

S5 Fig miRNA screening in human plasma RNA. The expression levels in human plasma of the 7 candidate circulating miRNAs from pig plasma. Mean C_q_ values are expressed after inter-plate calibration.
(TIF)

S6 Fig Evaluating the effect of biopsy mass on RNA yield and integrity. (A) RNA yield is constant over the range of biopsy masses from 1–6 μg. Linear regression *y* = −0.0161*x* + 1.326 (B) For biopsies weighing <1 mg, the yield is no longer constant; non-linear regression, least-squares fit *y* = 1.503*x*^−0.4715^ (C) The RIN remains constant across the range of biopsy masses.
(TIF)

S1 Data Raw data underlying all data figures are available in the zipped folder “S1_Data.zip” in csv format. (ZIP)
