# Supplementary material for: Optimisation of laboratory methods for whole transcriptomic RNA analyses in human left ventricular biopsies and blood samples of clinical relevance
Source: PLoS One. 2019 Mar 14;14(3):e0213685. doi: 10.1371/journal.pone.0213685 (PMC6417664; doi:10.1371/journal.pone.0213685)
Supplement: S1 File — An expanded methods section and Table B in S1 File can be found in the online supplement, “supplemental methods and results table.docx”. (DOCX) [file pone.0213685.s001.docx]

Supplemental methods

The ARCADIA (Association of non-coding RNAs with Coronary Artery Disease and type 2 Diabetes) study is an observational prospective cohort study that is supported as a portfolio study by the NIHR (National Institute for Health Research) and was developed across the Bristol Royal Infirmary (University Hospitals Bristol NHS Foundation Trust) and Hammersmith Hospital (Imperial College Healthcare NHS Trust). ARCADIA was designed to 1) characterise miRNA-driven expression changes in RNAs, proteins and metabolites in the left ventricle (LV) of patients with coronary artery disease (CAD)-induced myocardial ischaemia, 2) investigate how such changes are associated with and possibly induced by T2DM, and 3) to measure how these changes are mirrored by alterations in expression in biofluids. For the latter, we focus on those biofluids most influenced by the LV secretome, specifically the pericardial fluid (PF), a plasma ultrafiltrate that is in direct contact with the epicardium and the coronary circulation, and the transcoronary blood (collected from the ascending aorta supplying the coronary arteries and the coronary sinus where the blood exits the myocardium). The aim is to undertake network analysis of transcriptional signatures unique to each condition, and also to understand how the potential biomarkers of an altered cardiac status are trafficked from the LV to peripheral fluids that are clinically straightforward to sample.

ARCADIA has recruited 4 groups of cardiac surgery patients (Table S1). Samples obtained from these patients comprise left ventricular biopsies, pericardial fluid, peripheral and transcoronary blood, urine and (in CABG patients only) leftover saphenous vein.

| Group | IHD | T2DM | Hyp |
| --- | --- | --- | --- |
| 1. CABG-NDM | 1 | 0 | ? |
| 2. CABG-DM | 1 | 1 | ? |
| 3. AVR | 0 | 0 | 1 |
| 4. MVR | 0 | 0 | 0 |

The study consists of three stages. Stage 1 contains 12 patients from each surgical group, for characterization by RNA sequencing of small and long RNAs in the left ventricle tissue and of miRNA in biofluids and in extracellular vesicles extracted from the biofluids, by RT-qPCR screening and small RNA sequencing. Stage 2 contains a further 20 patients per surgical group (as defined in stage 1), and blood and urine samples from 20 non-diabetic and 20 diabetic volunteers not known to have cardiac disease. These samples are used to validate the findings from stage 1 (by RT-qPCR) in larger cohorts of patients. Stage 3 comprises the one-year post-surgical follow up of the CABG patients enrolled in Stages 1 and 2 and aims at identification of predictive biomarkers. The study protocol has been subsequently amended to include metabolomics and proteomics analyses on LV biopsies, biofluids and extracellular vesicles from all the patients.

**Table A.** By comparing patients undergoing coronary artery bypass graft (CABG) surgery with and without T2DM (CABG-DM and CABG-NDM respectively), with non-CAD patients without T2DM undergoing aortic valve replacement (AVR) surgery and non-CAD patients without T2DM undergoing mitral valve repair (MVR) surgery we will consider the effects of 3 disease features: ischaemic heart disease (IHD), type 2 diabetes mellitus (T2DM), hypertrophic and fibrotic remodelling (Hyp). The table indicates which features are present (1) or absent (0) in each group.

**Study participants**

For Stage 1, eligible participants have CAD and were undergoing first time isolated CABG using cold blood cardioplegia, or have aortic or mitral valvular disease (but no CAD) and were undergoing surgery for aortic valve replacement (AVR) or mitral valve repair (MVR). Patients in the AVR group represent a non-ischaemic control group. However, these patients present with cardiac hypertrophy and heart failure. Patients in the MVR group form a non-ischaemic and a non-hypertrophic control for the CAD/CABG and AVR groups. Hence, comparisons between the three groups will allow the influences of ischaemia and hypertrophy on RNA expression to be quantified (table 1).

For Stage 2, eligible participants are as for Stage 1 but also includes volunteers not known to have CAD or cardiovascular disease at large.

*Inclusion criteria*

Surgical participant may enter study at stage 1 or 2 if ALL the following apply:

1. Age between 40 and ≤80.

2. Patients undergoing CABG for CAD with cold blood cardioplegia and with a left ventricular ejection fraction ≥40%, or having aortic valve replacement (AVR) or mitral valve repair (MVR) for valvular disease and classified as NYHA class 1 or 2.

*Exclusion criteria*

Participant may not enter study if ANY of the following apply

1. Undergoing any emergency or salvage cardiac procedure
2. Had previous cardiac surgery
3. Has T1DM
4. Having ablation with the primary procedure
5. Has endocarditis at the time of the index admission
6. Undergoing mitral valve replacement
7. Undergoing aortic valve repair
8. Undergoing MVR but classified as NYHA class >2
9. Raised pre-operative Troponin

Non-surgical participant (stage 2 only) may enter study if:

1. Age between 40 and ≤80
2. No known CAD without T2DM OR

No known CAD with T2DM

**Study endpoints**

*Primary endpoint*

The primary endpoint for Stage 1 is quantified expression in myocardial biopsies and biofluids of RNAs including RNA modifications, determined by RNA sequencing. This stage will identify associations in disease specific molecular networks (T2DM, CAD and the combination) to identify nodal points which may represent potential therapeutic target for future intervention.

The primary endpoint for Stages 2 and 3 is quantified expression in myocardial biopsies and biofluids of candidate RNAs and RNA modifications selected at Stage 1 as being associated with T2DM, CAD or a combination of these characteristics.

The primary outcome for Stage 3 is expression of RNAs associated with specific adverse events.

*Secondary endpoints*

Secondary endpoints are expression of candidate RNAs in pericardial fluid, blood, urine and saphenous vein left over after surgery. Mechanisms of changes in RNA expression in the left ventricle and the release of RNAs from the myocardium into the blood will be investigated. Furthermore, proteomics and metabolomics analyses will be carried out and integrated together and with the RNA data using computational approaches. These analyses will provide a fully integrative model of the molecular signature of CAD patients and how this is influenced by T2DM.

**Statistical analysis**

Unpaired data sets were compared using the Kruskal-Wallis test, with Dunn’s correction for multiple comparisons. Due to the small sample size, the non-parametric Kruskal-Wallis test was chosen in preference to a one-way ANOVA. Paired data sets were compared using the Wilcoxon matched pairs signed rank test for ungrouped data, and t test with the Holm-Sidak correction for multiple testing for grouped data.

Supplemental Results Table

| Biopsy number | Total RNA (ng) | RIN |
| --- | --- | --- |
| 1 | 49.23 | 8.3 |
| 2 | 28.85 | 7.8 |
| 3 | 22.65 | 8 |
| 4 | 31.20 | 8.4 |
| 5 | 28.63 | 8.6 |
| 6 | 30.70 | N/A |
| 7 | 20.18 | 8.3 |
| 8 | 30.33 | 8.2 |
| 9 | 16.75 | 2.4 |
| 10 | 22.48 | 7.9 |
| 11 | 44.15 | 8.1 |
| 12 | 42.80 | 8.3 |
| 13 | 8.33 | 7.7 |
| 14 | 31.78 | 8.3 |
| 15 | 52.90 | 8 |
| 16 | 23.90 | 7.6 |
| 17 | 50.15 | 8.3 |
| 18 | 53.63 | 7.7 |
| 19 | 29.60 | 8.4 |
| 20 | 5.88 | 7.8 |
| 21 | 31.55 | 8 |
| 22 | 48.13 | 8.2 |
| 23 | 40.18 | 8.2 |
| 24 | 33.90 | 8.5 |
| 25 | 46.18 | 8.5 |
| 26 | 34.98 | 8.3 |
| 27 | 42.68 | 8.6 |
| 28 | 23.60 | 8.3 |
| 29 | 24.90 | 8.1 |
| 30 | 46.18 | 8.4 |
| 31 | 54.33 | 6.8 |
| 32 | 53.65 | 8 |
| 33 | 58.08 | 8 |
| 34 | 52.63 | 7.9 |
| 35 | 47.20 | 8.2 |
| 36 | 52.63 | 8 |
| 37 | 34.40 | 8.1 |
| 38 | 62.13 | 7.7 |
| 39 | 58.78 | 8.3 |
| 40 | 40.20 | 7.9 |
| 41 | 29.13 | 8.1 |
| 42 | 44.98 | 8.4 |
| 43 | 35.05 | 8.5 |
| 44 | 32.13 | 8.3 |
| 45 | 40.90 | 8.4 |
| 46 | 51.55 | 8.2 |
| 47 | 32.70 | 8.5 |
| 48 | 48.65 | 7.9 |
| 49 | 79.08 | 7.5 |
| 50 | 44.93 | 8.2 |
| 51 | 12.85 | 7.8 |
| 52 | 40.45 | 8 |
| 53 | 84.48 | 8.4 |
| 54 | 16.70 | 8 |
| 55 | 60.68 | 5.5 |
| 56 | 19.18 | 8.2 |
| 57 | 16.43 | 8.4 |
| 58 | 33.40 | 8.4 |
| 59 | 64.60 | 8.3 |
| 60 | 63.98 | 8.4 |
| 61 | 35.95 | 7.5 |
| 62 | 59.03 | 7.7 |
| 63 | 84.63 | 8.1 |
| 64 | 15.08 | 8.5 |
| 65 | 61.10 | 8.2 |
| 66 | 87.98 | 8.1 |
| 67 | 53.33 | 8 |
| 68 | 30.15 | 8.4 |
| 69 | 51.00 | 8.2 |
| 70 | 82.08 | 8.3 |
| 71 | 63.43 | 7.7 |
| 72 | 75.68 | 7.2 |
| 73 | 85.03 | 7.9 |
| 74 | 72.90 | 8.1 |
| 75 | 72.73 | 8.1 |
| 76 | 83.15 | 7.8 |
| 77 | 81.13 | 8.3 |
| 78 | 94.68 | 8 |
| 79 | 32.85 | 8.3 |
| 80 | 72.45 | 8.4 |
| 81 | 87.68 | 8 |
| 82 | 79.98 | 7.5 |
| 83 | 71.95 | 8.1 |
| 84 | 65.33 | 8.5 |
| 85 | 67.53 | 8.4 |
| 86 | 78.73 | 8 |
| 87 | 68.35 | 8.5 |
| 88 | 71.58 | 7.8 |
| 89 | 58.00 | 7.2 |
| 90 | 89.23 | 8.1 |
| 91 | 67.78 | 8.3 |
| 92 | 53.58 | 8.2 |
| 93 | 65.45 | 8.3 |
| 94 | 76.63 | 8.2 |
| 95 | 55.93 | 8.5 |
| 96 | 67.70 | 8.4 |

**Table B.** RNA yield and RIN from each human LV biopsy extracted with the modified *mir*Vana protocol. Biopsy mass 0.1-6 mg.
