# Supplementary figures and images for: Optimisation of laboratory methods for whole transcriptomic RNA analyses in human left ventricular biopsies and blood samples of clinical relevance
Source: PLoS One. 2019 Mar 14;14(3):e0213685. doi: 10.1371/journal.pone.0213685 (PMC6417664; doi:10.1371/journal.pone.0213685)

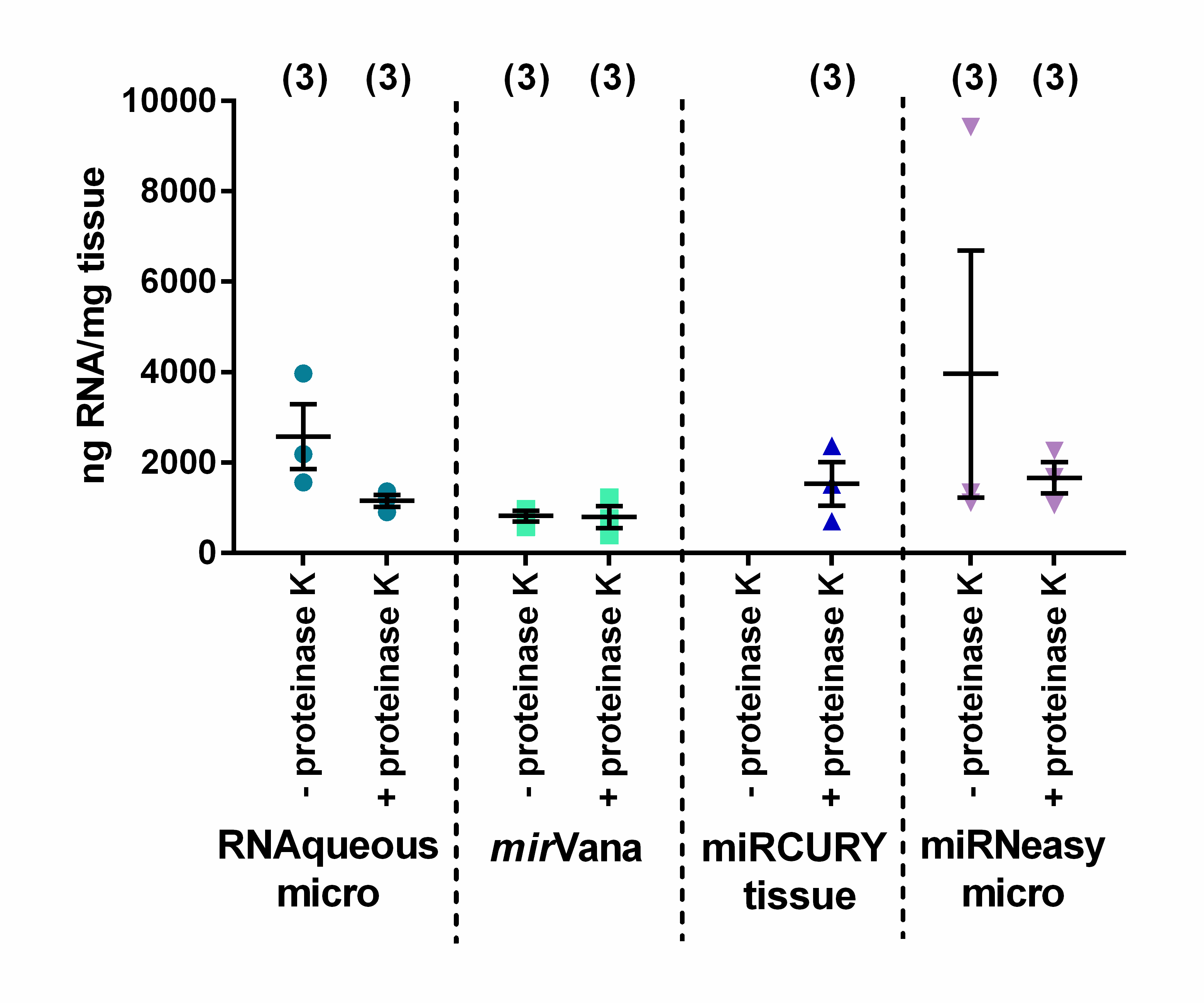

Supplement: S1 Fig — Bioanalyzer Nano chip quantification of LV biopsy RNA was similar to the spectrophotometry quantification presented in Fig 2A. RNA extracted according to each manufacturer’s protocol, with the addition of a proteinase K digestion in half the samples. n = 3 biopsies/experimental condition, mean±SEM. N.B. Exiqon miRCURY tissue protocol requires proteinase K, therefore no–proteinase K condition was done with this kit. Numbers in brackets indicate the number of samples in each group. (TIF) [file pone.0213685.s002.tif]

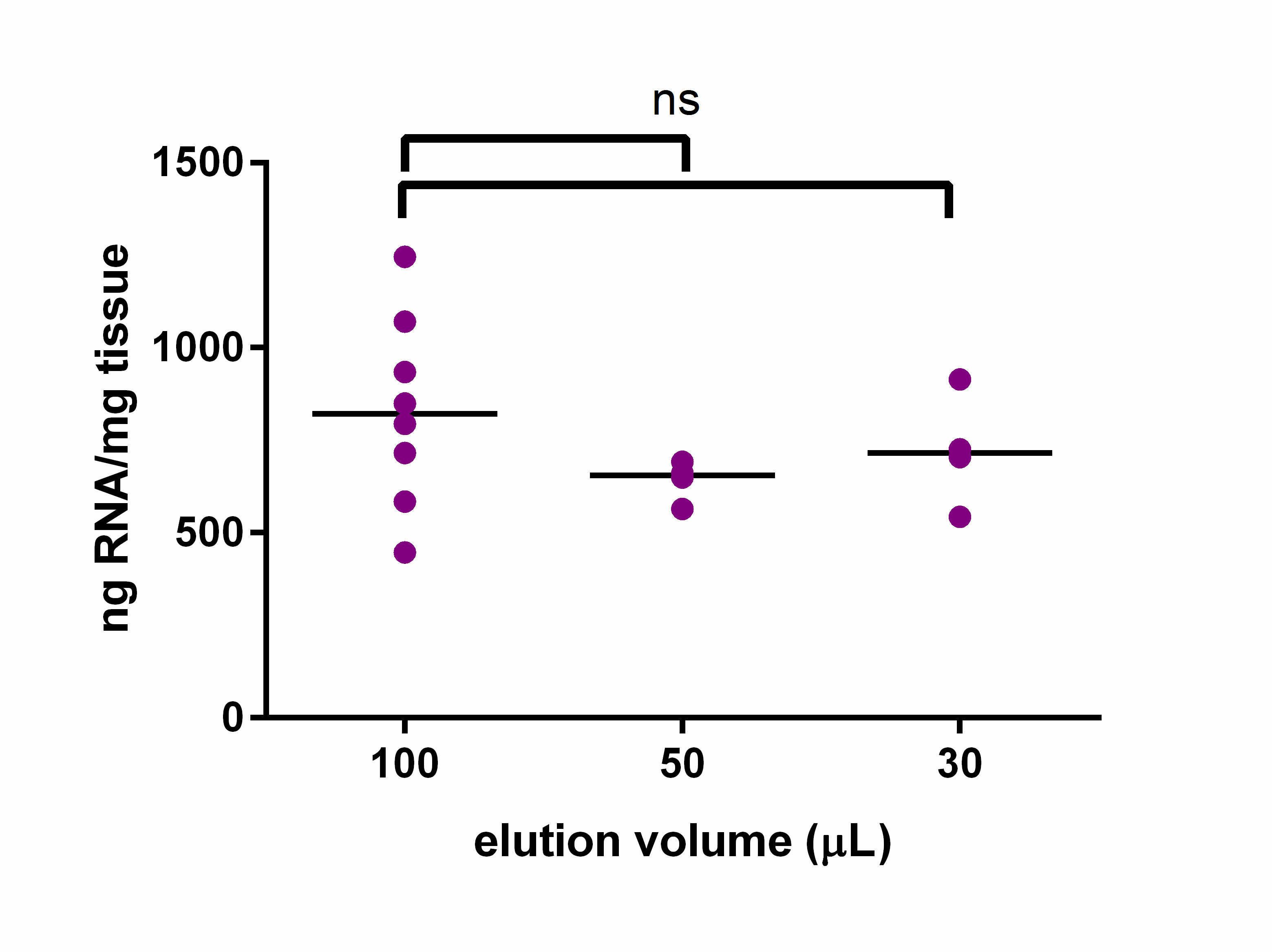

Supplement: S2 Fig — The mirVana protocol recommends elution in 100 μL water; with small tissue samples this can result in a sample too dilute for some downstream applications. Reducing the elution volume to 50 or 30 μL produced a more concentrated preparation without appreciable loss of RNA yield, measured by spectrophotometry (p = 0.2359, Kruskal-Wallis test). (TIF) [file pone.0213685.s003.tif]

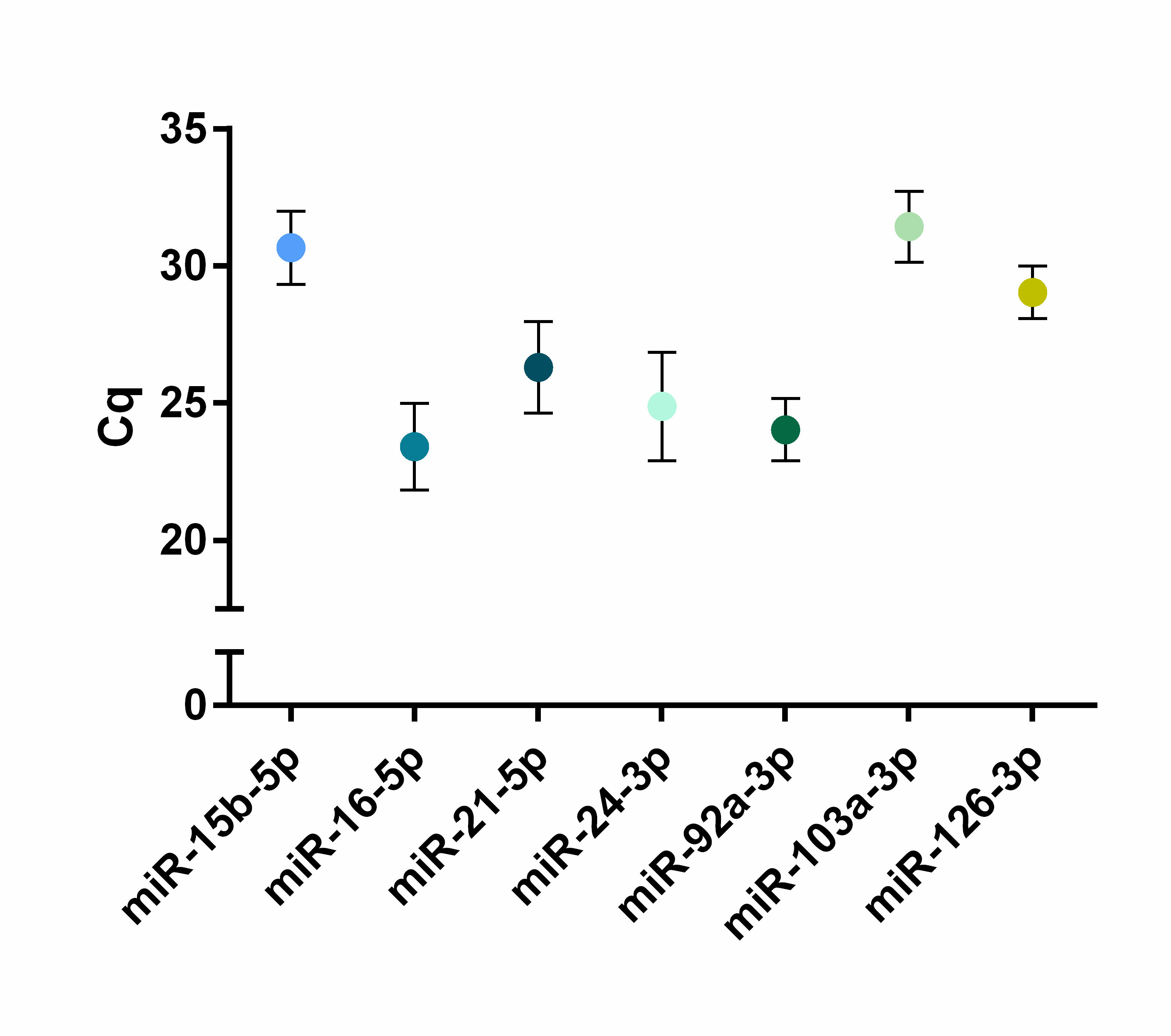

Supplement: S3 Fig — Two randomly selected pig plasma RNA samples were screened for the presence of 7 candidate circulating miRNAs predicted to be conserved between humans and pigs. (TIF) [file pone.0213685.s004.tif]

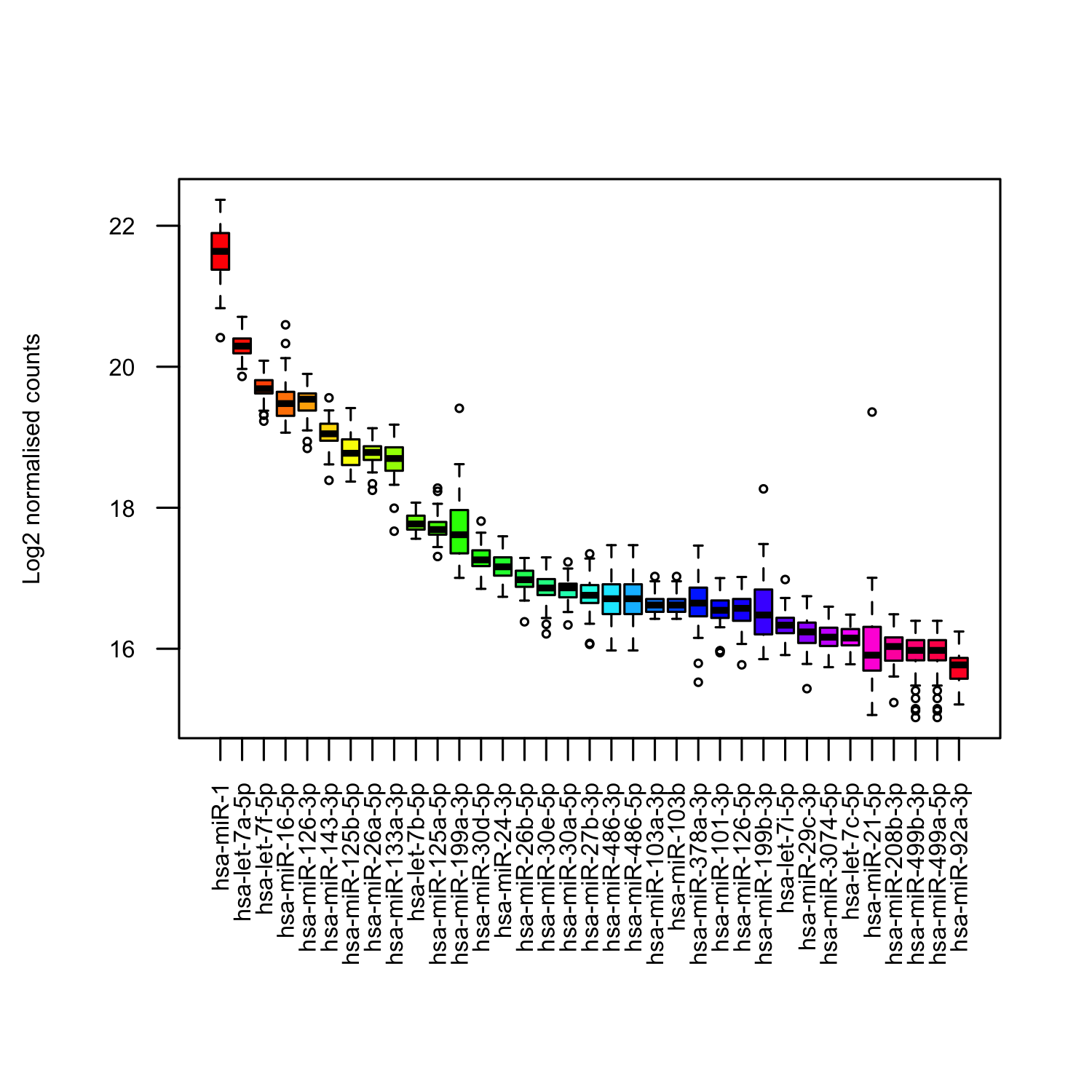

Supplement: S4 Fig — The expression levels of the top 35 miRNAs in LV biopsies are plotted as log2 normalised counts. For each plotted miRNA, the box indicates the upper (75%) and lower (25%) quartiles of the counts with a straight black line within the box indicating the median. The whiskers of the box indicate counts that lie outside of this. Circles represent outliers. All candidate circulating miRNAs from pig plasma are also expressed in human LV; only hsa-miR-15b-5p lies outside the top 35. (TIF) [file pone.0213685.s005.tif]

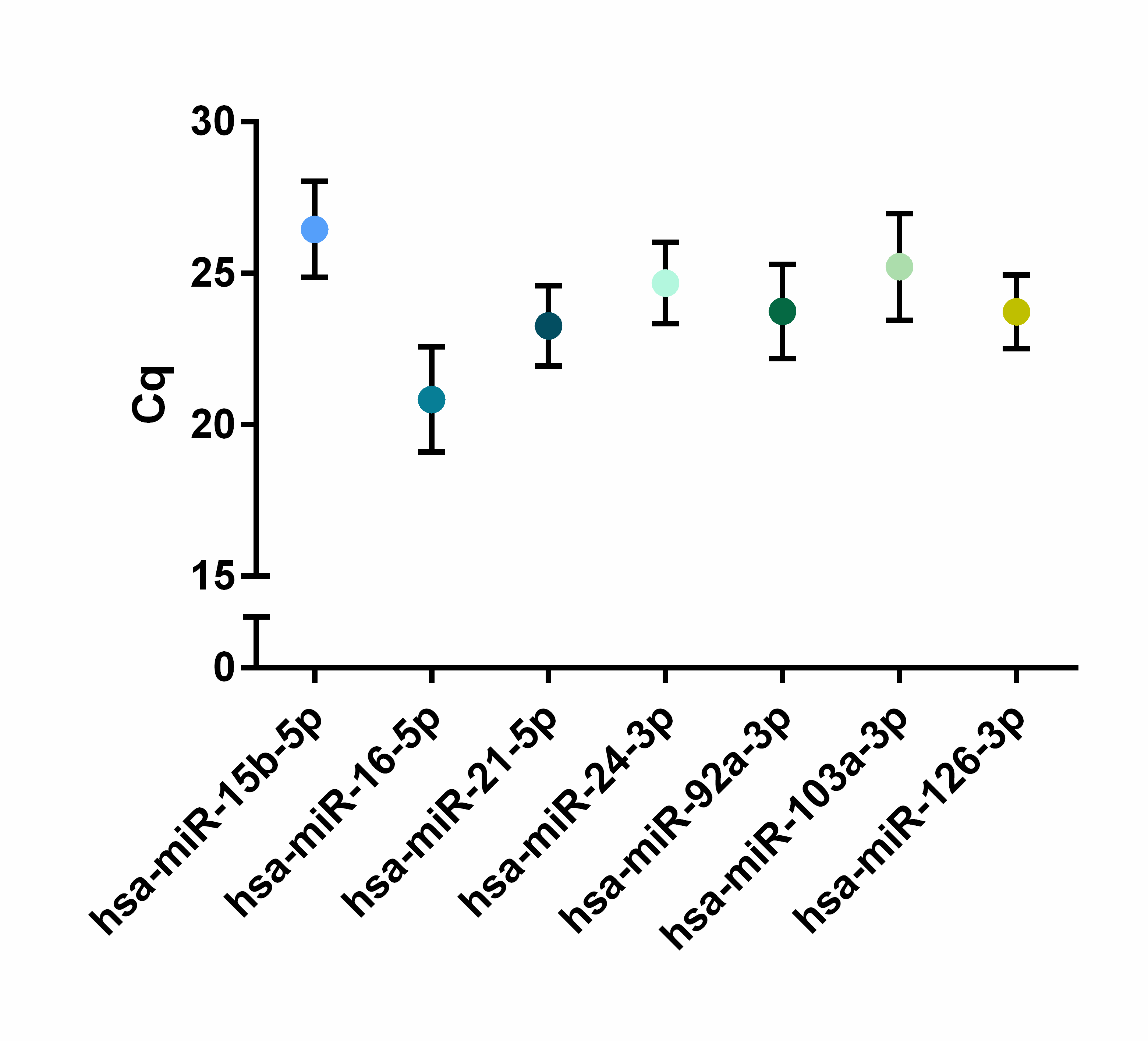

Supplement: S5 Fig — The expression levels in human plasma of the 7 candidate circulating miRNAs from pig plasma. Mean Cq values are expressed after inter-plate calibration. (TIF) [file pone.0213685.s006.tif]
